# Supplementary material for: Genome-wide CRISPR Screens in T Helper Cells Reveal Pervasive Crosstalk between Activation and Differentiation
Source: Cell. 2019 Feb 7;176(4):882–896.e18. doi: 10.1016/j.cell.2018.11.044 (PMC6370901; doi:10.1016/j.cell.2018.11.044)
Supplement: Data S2. Processed Data from All the Steps of the Analysis, Related to Figure 1 [file mmc2.zip › supplemental data/motif analysis/Pparg_homer/homerResults/motif8.similar.html]

motif8

## Information for motif8

A
C
G
T
A
T
G
C
G
C
A
T
C
G
A
T
G
C
T
A
A
G
C
T
A
T
G
C
G
C
A
T
A
T
C
G
T
G
C
A
  
Reverse Opposite:  

A
C
G
T
T
A
G
C
C
G
T
A
T
A
C
G
T
C
G
A
C
G
A
T
G
C
T
A
C
G
T
A
T
A
C
G
T
G
C
A
  

|  |  |
| --- | --- |
| p-value: | 1e-101 |
| log p-value: | -2.327e+02 |
| Information Content per bp: | 1.515 |
| Number of Target Sequences with motif | 1680.0 |
| Percentage of Target Sequences with motif | 13.42% |
| Number of Background Sequences with motif | 2798.9 |
| Percentage of Background Sequences with motif | 7.81% |
| Average Position of motif in Targets | 100.0 +/- 54.6bp |
| Average Position of motif in Background | 99.3 +/- 60.1bp |
| Strand Bias (log2 ratio + to - strand density) | 0.2 |
| Multiplicity (# of sites on avg that occur together) | 1.06 |
| Motif File: | file (matrix) reverse opposite |

### Similar de novo motifs found

|  |  |  |  |  |  |  |  |
| --- | --- | --- | --- | --- | --- | --- | --- |
| Rank | Match Score | Redundant Motif | P-value | log P-value | % of Targets | % of Background | Motif file |
| 1 | 0.947 | A C G T A T G C C G A T A C G T C G T A A C G T A G T C C G A T | 1e-96 | -221.485457 | 10.44% | 5.66% | motif file (matrix) |
| 2 | 0.640 | C G T A G T A C C T G A A T C G G T C A A C G T G T A C C G T A | 1e-51 | -118.868435 | 14.22% | 9.93% | motif file (matrix) |
| 3 | 0.715 | C G A T G T A C G C T A G A C T C G T A A C G T T G A C G C A T A T C G G C T A G C A T C T G A | 1e-38 | -87.781037 | 1.57% | 0.53% | motif file (matrix) |
| 4 | 0.790 | C G T A A C T G C G T A A C G T C G T A C T G A A T G C C G T A A C G T A G T C | 1e-34 | -78.761662 | 0.54% | 0.08% | motif file (matrix) |
